# Defect-Engineered MnO_2_@Ni Foam Electrode for Zinc-Ion Batteries Toward Mobile Robotics Applications

**DOI:** 10.3390/nano15171312

**Published:** 2025-08-26

**Authors:** Shilin Li, Dong Xie, Taoyun Zhou, Qiaomei Zhao, Muzhou Liu, Xinyu Li

**Affiliations:** 1 School of Information, Hunan University of Humanities, Science and Technology, Loudi 417000, China; lishilin09@huhst.edu.cn (S.L.); taoyun_2000@163.com (T.Z.); zhaoqiaomei@163.com (Q.Z.); liu_muzhou@foxmail.com (M.L.); 2 College of Physics and Electronic Information Engineering, Guilin University of Technology, Guilin 541004, China; lixinyu5260@163.com

**Keywords:** MnO_2_ electrode, oxygen vacancies, nickel foam substrate, energy storage for mobile robotics

## Abstract

Aqueous zinc-ion batteries (AZIBs) have gained significant attention as promising candidates for next-generation energy storage systems, especially in mobile robotics, due to their inherent safety, environmental friendliness, and low cost. However, the practical application of AZIBs is often hindered by slow Zn^2+^ diffusion and the poor structural stability of the cathode materials under high-rate or long-term operation. To address these challenges, a defect-engineered, binder-free MnO_2_ electrode, with a MnO_2_ loading of 1.35 mg·cm^−2^, is synthesized via in situ hydrothermal growth of ultrathin MnO_2_ nanosheets directly on a 3D conductive nickel foam scaffold, followed by reductive annealing to introduce abundant oxygen vacancies. These oxygen-rich defect sites significantly enhance Zn^2+^ adsorption, improve charge transfer kinetics, and contribute to enhanced pseudocapacitive behavior, further improving overall electrochemical performance. The intimate contact between the MnO_2_ and Ni substrate ensures efficient electron transport and robust structural integrity during repeated cycling. With this synergistic architecture, the MnO_2_@Ni electrode achieves a high specific capacity of 122.9 mAh·g^−1^ at 1 A·g^−1^, demonstrating excellent cycling durability with 94.24% capacity retention after 800 cycles and nearly 99% coulombic efficiency. This study offers a scalable strategy for designing high-performance, structurally stable Zn-ion battery cathodes with improved rate capability, making it a promising candidate for energy-intensive mobile robotic and flexible electronic systems.

## 1. Introduction

The rapid development of mobile robotics, autonomous systems, and next-generation smart sensing technologies has created pressing demands for energy storage devices that are not only energy-dense and power-efficient, but also inherently safe, cost-effective, and environmentally sustainable [1]. These requirements are particularly critical in dynamic, space-constrained, and thermally sensitive scenarios often encountered in robotic platforms. As the global energy storage market shifts toward decentralized and application-specific systems, identifying robust alternatives to conventional lithium-ion batteries (LIBs) has become an urgent research priority.

Although LIBs dominate the current market owing to their high energy density [2], their widespread deployment in mobile robotic applications is limited by inherent safety risks under thermal abuse, high material cost, and dependence on geopolitically constrained resources such as lithium and cobalt [3]. These limitations have spurred an interest in aqueous multivalent metal-ion batteries—especially aqueous zinc-ion batteries (AZIBs)—which are gaining recognition as safe and scalable energy storage systems for emerging flexible and intelligent electronics [4,5].

Zinc is naturally abundant, non-toxic, and exhibits a high theoretical capacity (819 mAh·g^−1^) and a relatively low redox potential (−0.762 V vs. SHE), making it well-suited for reversible Zn^2+^ plating/stripping in neutral aqueous electrolytes [6,7]. These properties make AZIBs particularly attractive for applications in portable electronics, wearable devices, and mobile robotics. However, the development of high-performance cathode materials remains a critical bottleneck toward realizing the full potential of ZIBs. Among various candidates, manganese dioxide (MnO_2_) has garnered substantial attention due to its low cost, environmental friendliness, and high theoretical capacity (~308 mAh·g^−1^) [8]. Nonetheless, MnO_2_ suffers from poor electrical conductivity and rapid capacity fading during cycling. To address these challenges, defect engineering—particularly the introduction of oxygen vacancies and cationic site modifications—has emerged as an effective strategy to modulate the electronic structure, enhance Zn^2+^ diffusion kinetics, and stabilize the cathode framework at the atomic scale [9,10].

In the pioneering work by Minakshi et al. [11], lithium insertion into manganese dioxide electrodes was demonstrated in aqueous Zn-MnO_2_ batteries. They explored the role of lithium intercalation in the structural stability and electrochemical performance of MnO_2_ in such battery systems. Their findings highlighted the potential of manganese dioxide to facilitate reversible charge storage processes, suggesting its promise for enhancing Zn^2+^ intercalation behavior as well. Later, Minakshi and Ionescu [12] also investigated the anodic behavior of zinc in Zn-MnO_2_ batteries using the ERDA technique, providing valuable insights into the electrochemical performance of these batteries, particularly in terms of Zn^2+^ dissolution and redox processes. These works laid the groundwork for the development of MnO_2_-based cathodes in zinc-ion batteries.

For example, ref. [13] showed that oxygen vacancies (V_O) significantly reduce the Gibbs free energy of Zn^2+^ adsorption, thereby enhancing ion transport and increasing the electrochemically active surface area. Simultaneously, these vacancies contribute to electron delocalization and improved conductivity [14]. Cationic defects, such as Mn-site vacancies, have also been shown to facilitate ion diffusion and increase redox-active sites [15].

In light of these findings, this work reports the design of a defect-engineered, binder-free MnO_2_ cathode prepared via in situ hydrothermal growth of ultrathin MnO_2_ nanosheets directly on a conductive nickel foam scaffold, followed by reductive annealing to introduce uniformly distributed oxygen vacancies. This dual-function strategy not only optimizes the intrinsic material properties of MnO_2_ but also ensures strong interfacial contact with the 3D conductive framework—effectively reducing charge transfer resistance, stabilizing the cathode structure, and enabling fast charge/discharge performance under demanding conditions.

The resulting MnO_2_@Ni electrode demonstrates a high specific capacity of 122.9 mAh·g^−1^ at 1 A·g^−1^ and exceptional cycling stability, maintaining 94.24% capacity retention after 800 cycles. Unlike conventional slurry-cast electrodes, the binder-free architecture minimizes interfacial resistance and mechanical degradation during cycling. Furthermore, the structural synergy between cationic defect engineering and the porous Ni foam scaffold enables improved electrolyte infiltration, enhanced Zn^2+^ diffusion kinetics, and superior rate capability. Overall, this study provides a scalable and practical strategy for developing high-performance MnO_2_-based cathodes for aqueous zinc-ion batteries. The proposed design holds great promise for energy-demanding applications such as mobile robotics, smart sensing systems, and other next-generation portable electronics.

## 2. Materials and Methods

### 2.1. Synthesis of MnO_2_@Ni

To prepare the MnO_2_@Ni electrode suitable for high-performance zinc-ion storage in mobile robotic applications, a hydrothermal method followed by controlled thermal treatment was employed. Specifically, 0.474 g of potassium permanganate (KMnO_4_) was dissolved in 60 mL of deionized water under magnetic stirring at room temperature for 10 min to obtain a uniform precursor solution. The solution was then transferred into a 100 mL polytetrafluoroethylene (PTFE)-lined stainless-steel autoclave.

Two pre-cleaned nickel foam substrates (each with a size of 3 × 3 cm^2^ and thickness of 1.5 mm) were immersed in the solution, followed by a hydrothermal reaction carried out at 180 °C for 4 h. Upon completion, the autoclave was cooled naturally to room temperature. The resulting samples were collected, rinsed thoroughly with deionized water to remove residual reactants, and dried overnight at 60 °C.

**Figure 1:**
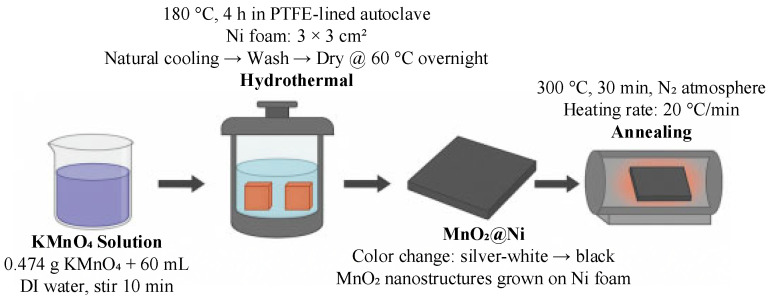
Schematic illustration of the fabrication process for the MnO_2_@Ni electrode.

After drying, the nickel foam changed color from silver-white to black, indicating the successful deposition of MnO_2_. Finally, the samples were annealed at 300 °C for 30 min under a nitrogen atmosphere (heating rate of 20 °C/min) to enhance crystallinity and structural stability. The fabrication process for the MnO_2_@Ni electrode is shown in Figure 1.

To evaluate the active material loading, the MnO_2_ mass deposited on the nickel foam was measured by subtracting the weight of the pristine Ni foam (before deposition) from the final weight after drying. The average mass loading of MnO_2_ on the Ni foam was calculated to be approximately 1.35 ± 0.02 mg·cm^−2^, which is within the range of low-loading electrodes commonly used for proof-of-concept evaluations.

### 2.2. Assembly of Binder-Free MnO_2_@Ni-Based AZIBs

To fabricate binder-free cathodes, the as-prepared MnO_2_@Ni electrodes—engineered with oxygen-rich defects—were directly employed as freestanding electrodes, eliminating the need for polymeric binders or conductive additives. In this configuration, the ultrathin MnO_2_ nanosheets are grown in situ on a 3D nickel foam scaffold, which acts simultaneously as the current collector and mechanical support. This design ensures excellent electrical conductivity, robust mechanical integrity, and intimate contact between the active material and substrate, thereby minimizing internal resistance and enhancing long-term stability.

Coin-type CR2016 AZIBs were assembled in an ambient atmosphere using the following components and procedure:

- Cathode: Binder-free MnO_2_@Ni electrode (circular, diameter: 16 mm)
- Anode: Zinc foil (diameter: 16 mm, mechanically polished before use)
- Electrolyte: Aqueous solution of 2 M ZnSO_4_ and 0.1 M MnSO_4_
- Separator: Whatman glass fiber membrane (diameter: 19 mm)
- Assembly procedure: Bottom cell case → MnO_2_@Ni cathode → 3 drops of electrolyte → separator → 3 additional drops of electrolyte → Zn anode → top cell case

The assembled coin cells were sealed using a precision crimping machine and allowed to rest for at least 16 h at room temperature to ensure complete electrolyte infiltration and electrode wetting before electrochemical testing. This simplified, binder-free cell architecture offers several advantages, including the elimination of toxic organic solvents (e.g., NMP), the avoidance of inert binder components (e.g., PVDF), and the reduction in inactive mass in the electrode. Furthermore, the integrated design improves energy density, structural compactness, and overall device safety—key features for advanced applications in space-constrained, flexible, or mobile robotic energy storage systems.

## 3. Results and Discussion

To systematically evaluate the zinc storage performance of the defect-engineered MnO_2_@Ni electrode as a freestanding cathode for AZIBs, especially in the context of mobile robotic applications, CR2016 coin-type cells were assembled under ambient conditions, as described in Section 2.2.

Electrochemical characterizations were conducted using a Neware battery testing system and an aqueous electrolyte composed of 2 M ZnSO_4_ and 0.1 M MnSO_4_. The addition of Mn^2+^ to the electrolyte serves not only to stabilize the MnO_2_ framework via redox buffering but also to suppress Mn dissolution during cycling, thereby improving long-term performance.

The electrochemical properties of the MnO_2_@Ni cathode were investigated using a combination of cyclic voltammetry (CV), galvanostatic charge–discharge (GCD) testing, and electrochemical impedance spectroscopy (EIS). All measurements were conducted within a potential window of 0.8–1.9 V vs. Zn/Zn^2+^, which is suitable for ensuring both capacity utilization and electrode stability.

These comprehensive evaluations were designed to probe key performance metrics including the following: rate capability, which reflects the electrode’s ability to deliver energy under varying power demands; cycling stability, which ensures operational durability over extended usage; charge transfer kinetics, which reveal the efficiency of interfacial electron and ion transport.

These characteristics are particularly important for real-time, high-power, and cyclic operations encountered in mobile robotics and autonomous energy systems. The integration of oxygen vacancy engineering with a 3D conductive scaffold is expected to address the key limitations of traditional MnO_2_-based cathodes, such as poor conductivity and structural degradation.

### 3.1. Characterization of MnO_2_@Ni

#### 3.1.1. Scanning Electron Microscopy (SEM) Analysis

In this study, scanning electron microscopy (SEM) was performed using a Bruker D8 model (Bruker Technology Co., Ltd., Beijing, China) operated at an accelerating voltage of 20 kV. Surface morphologies were captured using a secondary electron (SE) detector to ensure high-resolution imaging of the nanoscale features.

**Figure 2:**
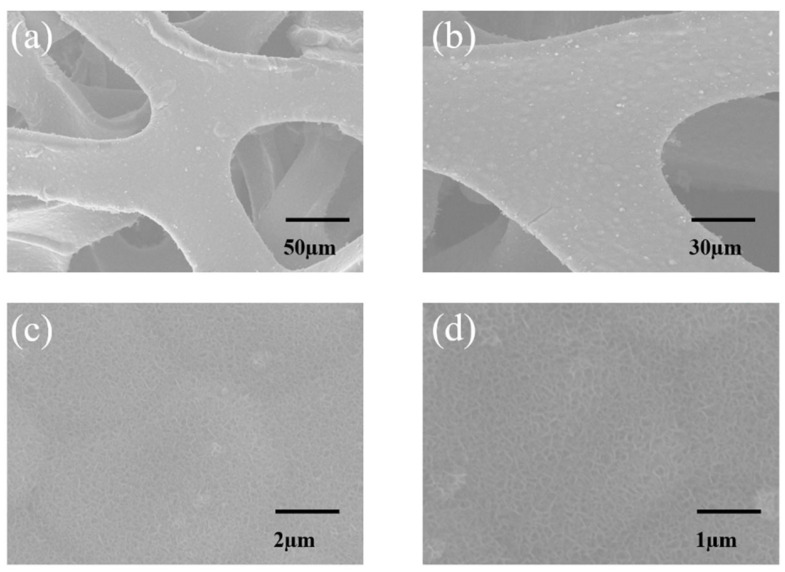
SEM Images. (**a**,**c**) are SEM images of the untreated sample. (**b**,**d**) are SEM images of the sample after 30 min of annealing.

The microstructure of MnO_2_@Ni before and after thermal treatment was examined, as shown in Figure 2, to assess morphological stability and structural integrity.

Figure 2a displays a low-magnification SEM image of the as-prepared MnO_2_@Ni sample, showing that the nickel foam skeleton is uniformly coated with a continuous MnO_2_ layer, without noticeable surface agglomeration. The scale bar of 50 μm highlights the 3D interconnected macroporous architecture of the Ni substrate, which serves as a mechanically robust and conductive framework for active material growth. At higher magnification in Figure 2c (scale bar 2 μm), the deposited MnO_2_ exhibits a highly dispersed and conformal ultrathin nanosheet morphology, with individual sheet-like features and an average lateral size of approximately 20–30 nm. This nanoscale texturing confirms successful nucleation and growth of 2D MnO_2_ structures with a high surface area, which is desirable for improving electrode–electrolyte interactions.

After annealing at 300 °C for 30 min, the microstructure remains well preserved, as seen in Figure 2b (low magnification, scale bar 30 μm) and Figure 2d (higher magnification, scale bar 1 μm). The nanosheet layer appears slightly thicker and denser, and the average feature size increases to approximately 25–40 nm, suggesting mild coarsening due to thermal treatment. Nevertheless, there is no observable collapse, aggregation, or detachment of the MnO_2_ coating, and the nanosheets remain firmly anchored to the Ni foam skeleton.

The observed morphological stability indicates that the thermal treatment improves crystallinity while maintaining the integrity of the nanosheet architecture. This robust structural retention is beneficial for achieving long-term cycling durability and enhanced electron/ion transport, which are essential for high-performance aqueous zinc-ion batteries.

#### 3.1.2. Transmission Electron Microscopy (TEM) Analysis

**Figure 3:**
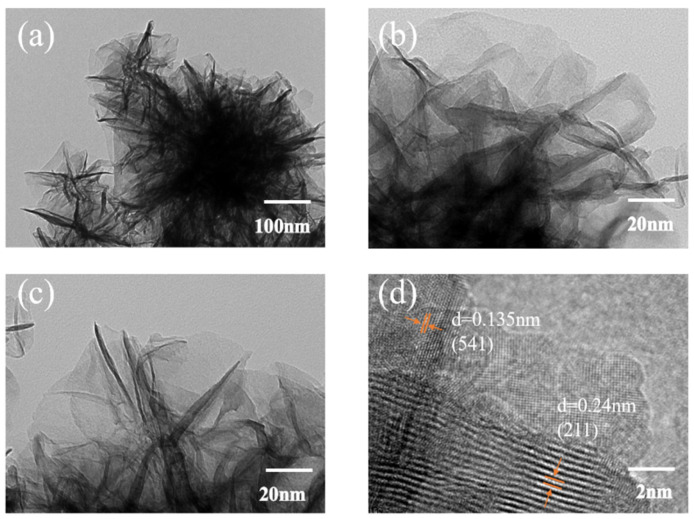
TEM Images. (**a**) is the low-magnification TEM image; (**b**,**c**) are the high-magnification TEM images; (**d**) is the HRTEM image.

To gain deeper insight into the nanoscale morphology and crystallinity of the defect-engineered MnO_2_@Ni electrode after thermal treatment, transmission electron microscopy (TEM) and high-resolution transmission electron microscopy (HRTEM) analyses were conducted. The corresponding images are presented in Figure 3.

As shown in the low-magnification TEM image in Figure 3a, the ultrathin sheet-like morphology of MnO_2_ nanosheets observed in SEM is well retained after annealing at 300 °C for 30 min. The two-dimensional structure remains intact without significant signs of aggregation or collapse, indicating the excellent thermal stability of the architecture. The nanosheets exhibit a flake-like morphology with irregular but continuous lateral edges. The lateral dimensions range from approximately 100 to 300 nm, while the thickness is estimated to be less than 5 nm, as inferred from the high electron transparency of the flakes. This ultrathin nature is beneficial for promoting short Zn^2+^ diffusion pathways and exposing a large number of electroactive sites.

Higher magnification TEM images in Figure 3b,c reveal a wrinkled and stacked nanosheet arrangement with a partially interconnected layered configuration. The absence of large particle-like agglomerates or dense grain boundaries suggests that the MnO_2_ is uniformly distributed and conformally coated onto the Ni foam framework. Such a configuration is expected to provide enhanced electrolyte accessibility, mechanical flexibility, and ion/electron transport—features essential for long-term cycling and fast-rate charge/discharge requirements in energy-demanding mobile robotic platforms.

The HRTEM image in Figure 3d displays clear and well-resolved lattice fringes with interplanar spacings of 0.24 nm and 0.135 nm, which are indexed to the (211) and (541) planes of MnO_2_, respectively. These findings are consistent with the XRD results and confirm the formation of a highly crystalline single-phase MnO_2_, most likely corresponding to α-MnO_2_. The absence of observable secondary phases or amorphous regions further supports the structural purity of the electrode.

The presence of such well-aligned crystalline domains in the nanosheet matrix is crucial for reducing internal resistance and ensuring efficient charge transfer during prolonged cycling. Moreover, the combination of high crystallinity with oxygen vacancy-rich surfaces offers a synergistic pathway for enhancing Zn^2+^ intercalation kinetics and improving the rate performance of the electrode.

#### 3.1.3. Diffraction and Raman Spectroscopy Analysis

**Figure 4:**
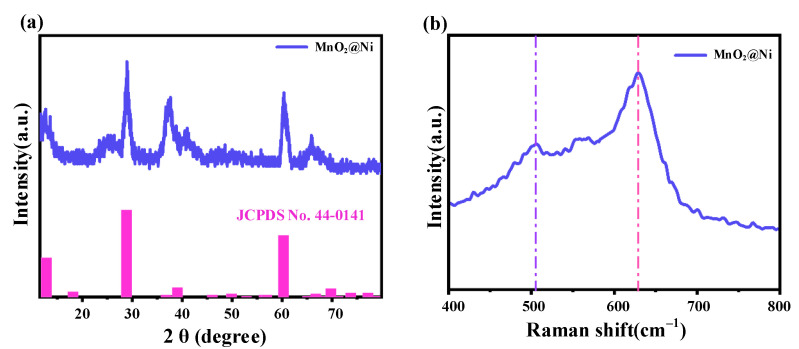
Analysis results of X-ray diffraction and Raman spectroscopy. (**a**) is the XRD pattern of the MnO_2_@Ni sample, (**b**) is the Raman spectrum of the MnO_2_@Ni sample.

To investigate the crystallographic phase, structural purity, and bonding environment of the MnO_2_@Ni electrode after annealing, X-ray diffraction (XRD) and Raman spectroscopy analyses were performed, and the results are presented in Figure 4. XRD measurements were conducted using a Bruker D8 Advance diffractometer (Bruker, Beijing, China) with Cu Kα radiation (λ = 1.5406 Å) operated at 40 kV and 40 mA. The data were collected over a 2θ range of 10–80°, providing comprehensive insights into the crystalline structure.

As shown in Figure 4a, the MnO_2_@Ni sample exhibits well-defined diffraction peaks located at 12.7°, 28.8°, 39.0°, and 60.2°, which can be indexed to the (110), (310), (330), and (521) crystal planes of α-MnO_2_ (JCPDS No. 44-0141). The absence of peaks corresponding to impurity phases such as Mn_3_O_4_ or MnO confirms the high phase purity of the synthesized material. Moreover, the narrow and sharp diffraction peaks indicate a high degree of crystallinity, which is consistent with the HRTEM observations presented in Figure 3d.

Although α-MnO_2_ is conventionally associated with a tunneled tetragonal crystal structure, the obtained MnO_2_ exhibits a two-dimensional (2D) layered nanosheet morphology, as confirmed by SEM and TEM analyses. This discrepancy can be attributed to the hydrothermal growth mechanism on the Ni foam, where anisotropic growth along specific crystal planes is driven by a combination of surface energy minimization and substrate-induced orientation. Similar morphological behavior has been previously reported in hydrothermally synthesized α-MnO_2_ systems, where nanosheet formation arises despite a tunneled crystal framework due to kinetic factors and nanoconfinement effects [16].

Raman spectroscopy in Figure 4b provides further confirmation of the structural characteristics. The strong peak centered at 632 cm^−1^ is attributed to the A_1_g symmetric stretching mode of Mn–O bonds in the MnO_6_ octahedra—characteristic of α-MnO_2_. Additionally, a broad band between 450 and 550 cm^−1^ corresponds to Mn–O–Mn bending vibrations. These features are consistent with prior reports on α-MnO_2_ [17].

Importantly, the slight redshift and broadening of the Raman bands compared to pristine α-MnO_2_ suggest the presence of lattice distortions and oxygen vacancies induced during the annealing process. Such defect structures are known to play a crucial role in enhancing the electrochemical activity of MnO_2_ by improving electronic conductivity, facilitating Zn^2+^ adsorption, and promoting ion diffusion kinetics [18,19]. These effects are particularly beneficial for high-performance AZIBs, where rapid and stable energy delivery is essential—especially in mobile robotics and other dynamic energy storage platforms.

#### 3.1.4. X-Ray Photoelectron Spectroscopy (XPS) Analysis

**Figure 5:**
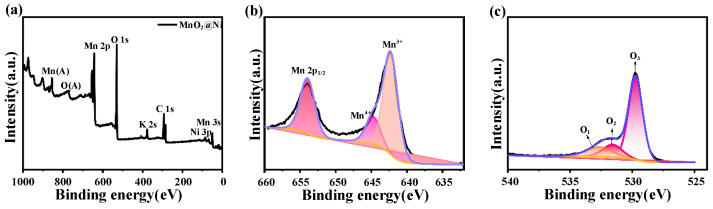
XPS results. (**a**) is the XPS spectrum of the MnO_2_@Ni sample; (**b**) is the high-resolution XPS spectrum of Mn 2p; (**c**) is the high-resolution XPS spectrum of O 1s.

To investigate the surface chemical composition and electronic states of the annealed MnO_2_@Ni electrode, X-ray photoelectron spectroscopy (XPS) analysis was conducted, and the results are presented in Figure 5.

The survey spectrum in Figure 5a displays the presence of nickel (Ni), manganese (Mn), oxygen (O), and carbon (C). The Ni signal originates from the underlying nickel foam substrate, while the C signal is likely due to residual organic contaminants or adventitious carbon during sample handling. A weak peak corresponding to potassium (K) is also detected, which can be attributed to trace remnants of the KMnO_4_ precursor, suggesting incomplete rinsing after hydrothermal synthesis. However, the K signal is of low intensity, and no other extraneous elements are observed, indicating the overall high chemical purity of the electrode material. Importantly, the trace presence of K^+^ is unlikely to significantly affect electrochemical performance, as it is presumed to be superficially adsorbed and electrochemically inactive under the tested conditions. Similar observations of K residuals have been reported in MnO_2_-based systems synthesized from KMnO_4_ and are generally considered benign when present at low concentrations.

The high-resolution Mn 2p spectrum shown in Figure 5b shows two well-defined peaks at 642.9 eV and 644.7 eV, corresponding to Mn^4+^ and Mn^3+^ oxidation states, respectively. The coexistence of these mixed valence states indicates a partial reduction of Mn^4+^ to Mn^3+^ during the annealing process and contributes to a higher density of redox-active sites. This redox duality facilitates reversible Mn^4+^/Mn^3+^ transitions, improves electronic conductivity, and enhances Zn^2+^ ion intercalation dynamics—all of which are crucial for delivering high-rate performance and long cycle life in aqueous zinc-ion batteries, especially in applications demanding reliable and responsive energy delivery such as mobile robotics.

The high-resolution O 1s spectrum in Figure 5c is deconvoluted into three distinct peak components, lattice oxygen (O_1_) located at 529.8 eV which is associated with Mn–O–Mn bonding in MnO_6_ octahedra, surface hydroxyl groups and adsorbed water species (O_2_) located at 531.2 eV, and oxygen vacancy-related species (O_3_) located at 531.8 eV. The appearance of the O_3_ peak reflects the formation of oxygen vacancies (V_O), which are introduced during annealing under a mildly reductive atmosphere. These vacancies are known to significantly influence the electronic structure of MnO_2_ by generating localized defect states that facilitate charge delocalization, enhance interfacial charge transfer, and reduce the energy barrier for Zn^2+^ diffusion. As a result, oxygen vacancies play a pivotal role in boosting the electrochemical activity and rate performance of the MnO_2_@Ni electrode.

Notably, the O_3_ peak exhibits higher intensity than the lattice oxygen peak (O_1_), which may seem counterintuitive at first. However, this observation is rationalized by the surface-sensitive nature of XPS, which probes only the top few nanometers of the material. In ultrathin, high-surface-area nanostructures—such as the MnO_2_ nanosheets synthesized in this study—oxygen vacancies tend to accumulate near the surface due to the thermodynamics of vacancy formation and diffusion during annealing. Consequently, their spectral contribution appears disproportionately large relative to the lattice oxygen, even if the absolute concentration in the bulk is lower. The presence of abundant surface oxygen vacancies thus strongly supports the enhanced Zn^2+^ transport kinetics and electrochemical responsiveness observed in subsequent battery performance tests.

### 3.2. Electrode Evaluation of the MnO_2_@Ni

To evaluate the zinc-ion storage capability of the binder-free MnO_2_@Ni electrode, CR2016 coin-type AZIBs were assembled in ambient air condition. The MnO_2_@Ni electrode served as the cathode, a pre-polished zinc foil was used as the anode, and a mixed aqueous electrolyte composed of 2 M ZnSO_4_ and 0.1 M MnSO_4_ was used to enhance cycling stability and suppress manganese dissolution.

All electrochemical characterizations were conducted at room temperature using a Neware battery testing system and a CHI 660E electrochemical workstation (CH Instruments, Shanghai, China). The working voltage window was set in the range of 0.8–1.9 V vs. Zn/Zn^2+^, which ensures reversible Zn^2+^ intercalation and stable electrochemical performance.

Electrochemical tests mainly included cyclic voltammetry (CV) performed at scan rates ranging from 5 to 100 mV·s^−1^ to analyze redox behavior, capacitive contribution, and kinetic response, galvanostatic charge–discharge (GCD) conducted at various current densities (from 0.1 A·g^−1^ to 5 A·g^−1^) to evaluate specific capacity, rate capability, and cycling durability and electrochemical impedance spectroscopy (EIS) conducted over a frequency range of 0.01 Hz to 10^5^ Hz using a sinusoidal perturbation of 5 mV amplitude. The resulting Nyquist plots were fitted using ZView 4.0 software, employing an equivalent circuit model with minimized fitting residuals. No IR compensation was applied.

This comprehensive electrochemical assessment confirms that the defect-engineered MnO_2_@Ni electrode delivers robust Zn^2+^ storage performance, combining high specific capacity, excellent rate capability, and long-term cycling stability. These attributes underscore its suitability for lightweight, flexible, and high-efficiency power sources, particularly in space-constrained and intermittently loaded systems such as autonomous robotics, wearable electronics, and intelligent sensing platforms.

#### 3.2.1. Cyclic Voltammetry (CV) Test

**Figure 6:**
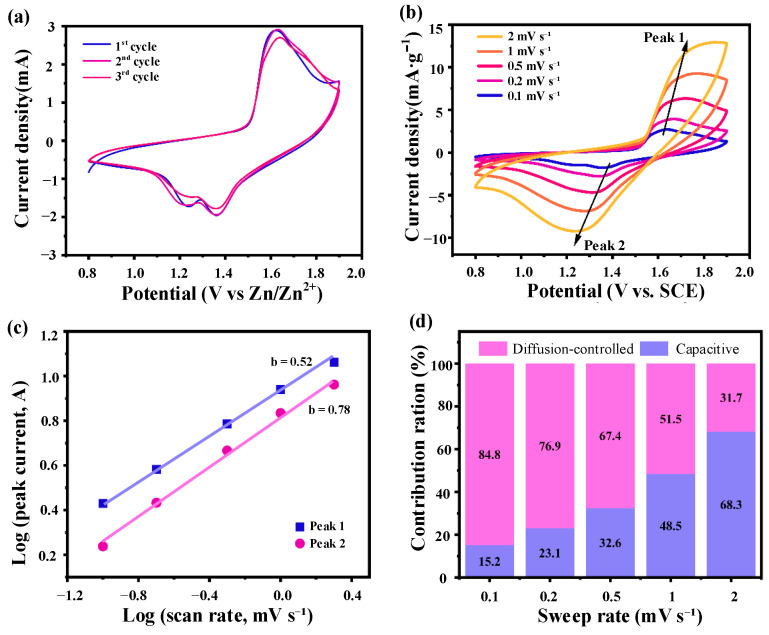
Electrochemical characteristics of the MnO_2_@Ni electrode. (**a**) is the first three CV curves of the MnO_2_@Ni electrode at a scan rate of 0.1 mV·s^−1^; (**b**) is the CV curves at different scan rates; (**c**) is the Log(i) vs. log(v) plot corresponding to the redox peaks from CV data; (**d**) is the contribution of capacitive-controlled and diffusion-controlled processes to the total current at different scan rates.

The electrochemical characteristics of the MnO_2_@Ni electrode were investigated by cyclic voltammetry (CV), and the corresponding results are presented in Figure 6.

As depicted in Figure 6a, the initial three CV cycles recorded at a scan rate of 0.1 mV·s^−1^ display two well-defined redox peaks centered at approximately 1.3 V (Zn^2+^ insertion) and 1.6 V (Zn^2+^ extraction) [20,21]. The high degree of overlap among the three cycles confirms excellent electrochemical reversibility and structural stability, indicating that the MnO_2_@Ni electrode can reliably support repeatable Zn^2+^ redox reactions during prolonged cycling. This is especially critical for real-time energy delivery in mobile robotic platforms that operate under dynamic and variable loading conditions.

Figure 6b shows CV profiles at scan rates ranging from 0.1 to 2.0 mV·s^−1^. As expected, the redox peak currents rise with the scan rate, and moderate peak separations are observed due to polarization effects. Nevertheless, the overall CV curves remain well-preserved across the scan rate range, indicating rapid ion diffusion, robust redox kinetics, and an excellent rate capability of the MnO_2_@Ni architecture.

To explore the charge storage mechanism, the relationship between peak current (*i*) and scan rate (*v*) was analyzed using the power-law equation [22]:

(1) $$i=av^{b}$$

where *i* is the peak current (A), *v* is the scan rate (V·s^−1^), *a* is an empirical fitting constant that depends on factors such as the electrochemical behavior, structural characteristics, and electrode surface area of the specific material. It is not a fixed theoretical value but is determined through fitting experimental data. *b* is the slope that characterizes the charge storage mechanism. The *b*-value can be used to differentiate between capacitive and diffusion-controlled processes.

Taking the logarithm of both sides yields

(2) logi=blogv+log(a)

The slope *b* provides insight into the charge storage kinetics: *b* ≈ 0.5 indicates a diffusion-controlled intercalation process, while *b* ≈ 1.0 implies a surface-dominated capacitive mechanism. As shown in Figure 6c, the calculated *b*-values for the anodic and cathodic peaks are 0.52 and 0.78, respectively, suggesting a hybrid energy storage mechanism in which both diffusion-controlled and pseudocapacitive processes contribute significantly.

To further distinguish the relative contributions of these two mechanisms, the semi-empirical method proposed by Dunn et al. was employed [22], where the total current (*i*) at a given potential follows

(3) $$i=k_{1}v+k_{2}\sqrt{v}$$

Here, the first term $\left(k_{1}v\right)$ represents the capacitive contribution, while the second term $\left(k_{2}\sqrt{v}\right)$ corresponds to the diffusion-controlled contribution. Rearranging gives

(4) $$\frac{i}{\sqrt{v}}=k_{1}\sqrt{v}+k_{2}$$

By plotting $\frac{i}{\sqrt{v}}$ versus $\sqrt{v}$, the values of *k*_1_ and *k*_2_ can be obtained, allowing the quantitative separation of the capacitive and diffusion-controlled contributions at various scan rates.

As illustrated in Figure 6d, the capacitive contribution increases markedly with increasing scan rate. At 0.1 mV·s^−1^, the diffusion-controlled process dominates (∼84.8%), indicative of bulk Zn^2+^ intercalation into MnO_2_. However, at 2.0 mV·s^−1^, the capacitive contribution rises significantly to ∼68.3%, highlighting a transition to surface-controlled pseudocapacitive behavior under fast scanning conditions. This transition reflects the material’s kinetic adaptability, which is essential for high-power energy storage.

These findings collectively indicate that the MnO_2_@Ni electrode exhibits a hybrid charge storage mechanism, where battery-type intercalation and surface-driven capacitive reactions coexist. The ultrathin 2D nanosheet structure and the conductive 3D Ni foam scaffold synergistically promote efficient Zn^2+^ transport and rapid redox kinetics. Such a mechanism offers the dual benefits of high energy density and high power density, making the MnO_2_@Ni electrode highly suitable for next-generation applications in flexible, fast-charging, and space-constrained systems, such as mobile robotics, wearable electronics, and autonomous sensing devices.

#### 3.2.2. Galvanostatic Charge–Discharge (GCD) Test

**Figure 7:**
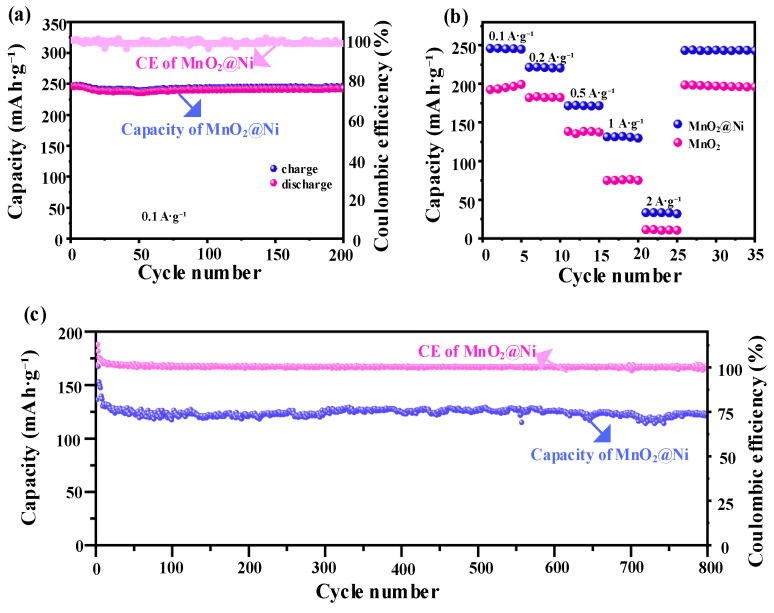
Galvanostatic charge–discharge performance of the electrodes. (**a**) Cycling performance of the MnO_2_@Ni electrode over 200 cycles at a current density of 0.1 A·g^−1^; (**b**) rate capability comparison between MnO_2_@Ni and pristine MnO_2_ electrodes at various current densities; (**c**) long-term cycling stability of the MnO_2_@Ni electrode at a current density of 1 A·g^−1^ over 800 cycles.

To further evaluate the electrochemical performance of the MnO_2_@Ni electrode as a freestanding cathode for AZIBs, galvanostatic charge–discharge (GCD) tests were performed at various current densities, as shown in Figure 7. These tests aim to assess the specific capacity, rate capability, and long-term cycling stability under different operational conditions.

As shown in Figure 7a, the MnO_2_@Ni electrode demonstrates excellent short-term cycling stability at a low current density of 0.1 A·g^−1^, maintaining a stable discharge capacity of approximately 246 mAh·g^−1^ over 200 cycles with negligible capacity degradation. This high retention reflects excellent electrochemical reversibility and structural robustness, even under extended operation. The GCD curves at 0.1 A·g^−1^ exhibit quasi-plateau regions centered near ~1.3 V (discharge insertion) and ~1.6 V (charge extraction), which are consistent with the redox peaks observed in cyclic voltammetry (CV) and indicate a dominant battery-type Zn^2+^ intercalation coupled with Mn^4+^/Mn^3+^ redox. This bulk intercalation behavior correlates with the *b*-value of 0.52 derived from CV analysis, signifying a diffusion-controlled process at low current densities.

The rate performance of the electrode, depicted in Figure 7b, further demonstrates its exceptional kinetic adaptability. The MnO_2_@Ni electrode delivers specific capacities of 247, 220, 170, 125, and 35 mAh·g^−1^ at current densities of 0.1, 0.2, 0.5, 1.0, and 2.0 A·g^−1^, respectively. Notably, when the current density returns from 2.0 A·g^−1^ to 0.1 A·g^−1^, the capacity nearly recovers to its original value, confirming the excellent rate capability and structural integrity of the electrode. As the current density increases, the GCD curves lose sharp plateaus and become more sloping, which aligns with the increasing capacitive contribution observed in the CV analysis (to ~68.3% at 2.0 mV·s^−1^). This transition from a plateau to a slope indicates the shift towards a surface-controlled pseudocapacitive process at higher rates, driven by the ultrathin nanosheet morphology and abundant oxygen vacancies that accelerate Zn^2+^ adsorption and interfacial charge transfer. In contrast, pristine MnO_2_ electrodes show significantly inferior capacity retention under identical testing conditions, which underscores the effectiveness of the integrated design strategy adopted for MnO_2_@Ni.

In terms of long-term cycling durability, the MnO_2_@Ni electrode clearly outperforms traditional MnO_2_-based cathodes derived from electrolytic manganese dioxide (EMD). For example, Minakshi et al. [21] studied lithium intercalation in Zn|MnO_2_|aqueous LiOH cells using both EMD and battery-grade MnO_2_, reporting an initial capacity of ~180 mAh·g^−1^, which deteriorated rapidly due to structural degradation and Mn dissolution in aqueous media.

By contrast, the MnO_2_@Ni electrode in this work not only achieves a higher initial capacity (~246 mAh·g^−1^ at 0.1 A·g^−1^) but also demonstrates remarkable cycling stability under harsh conditions. As shown in Figure 7c, it retains approximately 92.24% of its capacity after 800 continuous cycles at a high current density of 1 A·g^−1^, with a coulombic efficiency consistently maintained near 99%, highlighting the superior reversibility of Zn^2+^ insertion/extraction and minimal parasitic reactions. The GCD profile at 1 A·g^−1^ further reveals the electrode’s ability to sustain a significant portion of its capacity even under high-rate cycling, with a consistently high coulombic efficiency. This exceptional electrochemical performance is attributed to the synergistic design of the electrode structure and chemical composition. The in situ growth of ultrathin MnO_2_ nanosheets directly onto the 3D conductive Ni foam scaffold ensures enhanced electronic conductivity and mechanical resilience, strong interfacial contact between the active material and substrate and uniform dispersion of MnO_2_, which minimizes agglomeration and structural collapse during cycling.

In addition, the oxygen vacancy engineering introduced via reductive annealing plays a critical role by increasing the number of electrochemically active sites, facilitating fast Zn^2+^ diffusion and interfacial redox reactions, and promoting pseudocapacitive behavior, which further enhances rate capability. The combination of these factors facilitates a fast Zn^2+^ intercalation at low current densities and a fast surface-controlled pseudocapacitive reaction at high rates.

Compared with other reported MnO_2_-based composites—such as MnO_2_/rGO/PANI hybrids, α-MnO_2_, and MnO_2_@C electrodes [16,23,24,25]—the MnO_2_@Ni electrode achieves superior rate performance and long-term cycling stability. This validates the efficacy of the dual optimization strategy combining defect engineering and conductive substrate integration, effectively addressing the traditional limitations of MnO_2_ cathodes, including poor electrical conductivity and structural instability.

#### 3.2.3. Electrochemical Impedance Spectroscopy (EIS) Test

**Figure 8:**
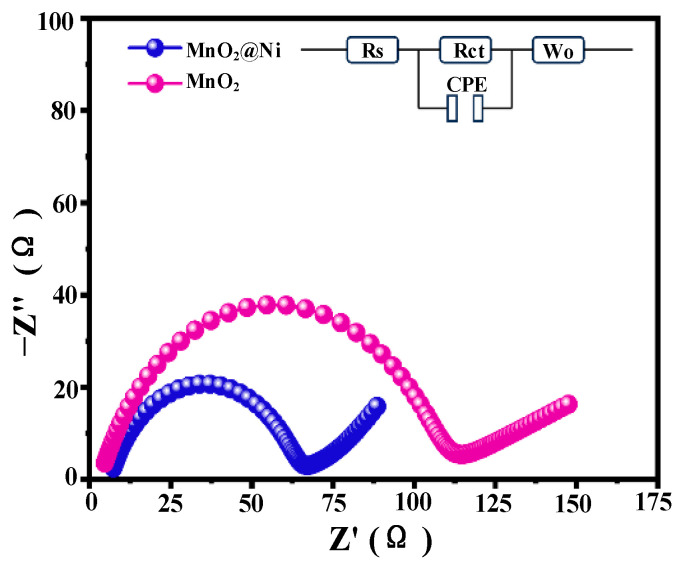
Impedance comparison of MnO_2_@Ni and MnO_2_.

Electrochemical impedance spectroscopy (EIS) was conducted to evaluate the charge transfer kinetics and Zn^2+^ diffusion behavior of the MnO_2_@Ni electrode, with the Nyquist plots illustrated in Figure 8. The impedance spectra consist of a depressed semicircle in the high-to-medium frequency region—attributed to the charge transfer resistance (Rct)—and a straight line at low frequencies, which corresponds to the Warburg impedance (Wo) associated with ion diffusion processes.

Compared to pristine MnO_2_, the MnO_2_@Ni electrode demonstrates a significantly smaller Rct (~63 Ω), as derived from equivalent circuit fitting. In contrast, the R_ct of the pristine MnO_2_ electrode is markedly higher at approximately 110 Ω. This reduction confirms that the integration of ultrathin MnO_2_ nanosheets with the 3D conductive Ni foam scaffold markedly improves interfacial charge transfer kinetics. The interconnected porous architecture of the Ni foam provides continuous electron conduction pathways and ensures intimate contact between the active material and current collector, effectively lowering the energy barrier for electron and ion transport.

In addition to Rct, the series resistance (Rs)—which includes contributions from the intrinsic resistance of the electrode and the electrolyte—was slightly lower for the MnO_2_@Ni electrode (5.0 Ω) than for pristine MnO_2_ (5.7 Ω). This further supports the enhanced electrical conductivity and improved electrode–electrolyte interface compatibility imparted by the Ni foam framework.

The constant phase element (CPE) parameters extracted from the fitting provide insight into the electrode’s capacitive behavior. For MnO_2_@Ni, the CPE-T and CPE-P values were determined to be 3.10 × 10^−4^ and 0.790, respectively, approaching the behavior of an ideal capacitor (P → 1). In comparison, the pristine MnO_2_ electrode exhibits slightly inferior values (CPE-T = 2.40 × 10^−4^, *p* = 0.765), indicative of less efficient double-layer formation and charge accumulation at the electrode–electrolyte interface.

In the low-frequency domain, the MnO_2_@Ni electrode presents a more vertical line (steeper slope), indicative of enhanced Zn^2+^ diffusion and a more prominent pseudocapacitive charge storage mechanism. The Warburg coefficients Wo-R and Wo-T were calculated to be 78.0 Ω and 66.0 s, respectively, for MnO_2_@Ni, significantly lower than those of the pristine MnO_2_ electrode (125.0 Ω and 85.0 s). These reductions confirm more facile ion transport through the porous electrode structure and suggest that the introduction of oxygen vacancies and nanoscale architecture effectively suppresses diffusion impedance.

The full set of electrochemical impedance parameters is summarized in Table 1, clearly demonstrating that the MnO_2_@Ni electrode exhibits lower charge transfer and series resistances, superior capacitive characteristics, and enhanced Zn^2+^ diffusion dynamics.

### 3.3. Comparative Performance Analysis

To comprehensively evaluate the electrochemical performance of the MnO_2_@Ni hybrid electrode, a multi-dimensional comparative analysis was performed against a series of representative MnO_2_-based composite systems reported in [7,9,10,15,20,26,27,28,29]. Key evaluation criteria included rate capability, cycling stability, and interfacial charge transfer characteristics, with particular attention to the influence of material architecture and electrolyte formulation. The reference systems encompass various MnO_2_/carbon and MnO_2_/conducting polymer composites, synthesized through diverse methods and operated in either pure ZnSO_4_ electrolytes or ZnSO_4_ supplemented with MnSO_4_. Notably, the incorporation of Mn^2+^ ions has been reported to contribute to redox buffering and phase stabilization, thereby positively affecting overall cycling performance.

While the MnO_2_@C core–shell structure [7], γ-MnO_2_/graphene composite [9], and Mn_3_O_4_/CNTs [15] enhance electrochemical performance through carbon modification or defect engineering, they still experience partial capacity fading or rely on complex synthesis processes. The Cu–MnO_2_·nH_2_O cathode [10] delivers a high initial capacity but is limited by phase transformations during cycling. In contrast, the MnO_2_@Ni hybrid electrode in this study integrates the direct in situ growth of ultrathin MnO_2_ nanosheets on a 3D conductive Ni foam scaffold with oxygen vacancy engineering, which ensures strong interfacial contact, abundant active sites, and fast charge transfer. This unique combination results in a high reversible capacity (246 mAh·g^−1^ at 0.1 A·g^−1^), excellent rate performance, and superior cycling stability (94.24% retention after 800 cycles at 1.0 A·g^−1^, CE ≈ 99%), distinguishing this work from previous studies. A detailed summary of the electrochemical metrics is provided in Table 2.

## 4. Conclusions

In this study, a novel synergistic design strategy integrating oxygen defect engineering and a three-dimensional (3D) conductive nickel foam scaffold was developed to construct a high-performance MnO_2_-based cathode for AZIBs. The MnO_2_@Ni electrode with a MnO_2_ loading of 1.35 mg·cm^−2^ was successfully fabricated via in situ hydrothermal growth of ultrathin MnO_2_ nanosheets directly on nickel foam, followed by reductive annealing to introduce uniformly distributed oxygen vacancies. This innovative hierarchical architecture significantly improves electronic conductivity, provides abundant electroactive sites for Zn^2+^ adsorption, and facilitates rapid ion transport through its interconnected porous network. Structural and spectroscopic characterizations confirmed the formation of a highly crystalline and oxygen-deficient MnO_2_ phase, which is critical for improving electrochemical performance. Electrochemical analyses demonstrated excellent performance metrics, including a high specific capacity of 122.9 mAh·g^−1^ at 1 A·g^−1^, long-term cycling stability with 94.24% capacity retention after 800 cycles, and nearly 99% coulombic efficiency. Notably, the electrode also exhibited a reduced charge transfer resistance of approximately 63 Ω, highlighting its superior interfacial charge transport properties. These results demonstrate the robust electrochemical reversibility, enhanced ion/electron kinetics, and durability of the MnO_2_@Ni electrode, showcasing its promising potential for high-power and high-efficiency energy storage applications, particularly in mobile robotics, flexible electronics, and next-generation sustainable power systems. This work stands apart from previous studies by combining defect engineering and direct, in situ synthesis on a 3D conductive substrate, leading to significant improvements in the electrochemical stability and energy storage performance, making this approach highly promising for future energy storage systems.

## Figures and Tables

**Table 1:** Summary of EIS fitting parameters for MnO_2_@Ni and MnO_2_ electrodes.

Sample	Rs(Ω)	Rct(Ω)	CPE-T	CPE-P	Wo-R(Ω)	Wo-T(s)	Wo-P
MnO_2_@Ni	5.0	63.0	3.10 × 10^−4^	0.790	78.0	66.0	0.460
MnO_2_	5.7	110.0	2.40 × 10^−4^	0.765	125.0	85.0	0.460

**Table 2:** Comparative analysis of electrochemical performance of selected MnO_2_-based electrode materials.

Materials	Mass Loading (mg/cm^−2^)	Current Density (A/g)	Specific Capacity (mAh/g)	Cycling Stability	Ref.
MnO_2_@C core–shell (carbon coating)	~1–2	0.1	210	102 mAh·g^−1^ after 600 cycles at 0.8 A·g^−1^	[7]
γ-MnO_2_/Graphene composite	~1	0.5	301	95.8 mAh·g^−1^ at 10 A·g^−1^, excellent rate	[9]
Cu–MnO_2_·nH_2_O (from Cu–MnO transformation)	~1–2	0.1	320	>70% after 1000 cycles	[10]
Mn_3_O_4_/CNTs (defect engineered)	1–2	0.1	420.6	84.1% after 2800 cycles at 2.0 A·g^−1^	[15]
γ-MnO_2_(EMD type)	<1	0.5	~100–180	Rapid decay, poor retention	[20]
MnO_2_@Ni	1.35	0.1	246	94.24% after 800 cycles at 1.0 A·g^−1^; CE ≈ 99%	This work

## Data Availability

All the datasets used in this manuscript are publicly available datasets already in the public domain.
